# Predicting Essential Metabolic Genome Content of Niche-Specific Enterobacterial Human Pathogens during Simulation of Host Environments

**DOI:** 10.1371/journal.pone.0149423

**Published:** 2016-02-17

**Authors:** Tong Ding, Kyle A. Case, Morrine A. Omolo, Holly A. Reiland, Zachary P. Metz, Xinyu Diao, David J. Baumler

**Affiliations:** 1 Department of Food Science and Nutrition, University of Minnesota-Twin Cities, St. Paul, Minnesota, United States of America; 2 Microbial and Plant Genomics Institute, University of Minnesota-Twin Cities, St. Paul, Minnesota, United States of America; 3 Biotechnology Institute, University of Minnesota-Twin Cities, St. Paul, Minnesota, United States of America; University of Louisville, UNITED STATES

## Abstract

Microorganisms have evolved to occupy certain environmental niches, and the metabolic genes essential for growth in these locations are retained in the genomes. Many microorganisms inhabit niches located in the human body, sometimes causing disease, and may retain genes essential for growth in locations such as the bloodstream and urinary tract, or growth during intracellular invasion of the hosts’ macrophage cells. Strains of *Escherichia coli* (*E*. *coli*) and *Salmonella* spp. are thought to have evolved over 100 million years from a common ancestor, and now cause disease in specific niches within humans. Here we have used a genome scale metabolic model representing the pangenome of *E*. *coli* which contains all metabolic reactions encoded by genes from 16 *E*. *coli* genomes, and have simulated environmental conditions found in the human bloodstream, urinary tract, and macrophage to determine essential metabolic genes needed for growth in each location. We compared the predicted essential genes for three *E*. *coli* strains and one *Salmonella* strain that cause disease in each host environment, and determined that essential gene retention could be accurately predicted using this approach. This project demonstrated that simulating human body environments such as the bloodstream can successfully lead to accurate computational predictions of essential/important genes.

## Introduction

Computational modeling has been widely used as an efficient approach in microbiology, which introduces mathematical components including variables, parameters, and equations in network constructions to reflect the behavior of organisms. Numerous types of networks have been constructed including signaling, regulatory, and metabolic pathways for organisms ranging from microorganisms, such as *E*. *coli*, to multi-cellular eukaryotic organisms. By constructing genome-scale metabolic models (GEMs), the nature of an organism can be explored through computational analysis of its genome content. The *E*. *coli* K-12 strain MG1655 has had extensive computational metabolic networks generated for it so far, and its existing models are quite advanced that contain >2,000 reactions, >1,000 genes, and >1,000 metabolites [1–6]. These genome-scale models have been used for many studies that have guided the engineering of strains for increasing valuable end-products, promoting enzyme discovery, providing insight into the genome evolution of other enterobacteria [7,8], and leading to a new understanding of the connectivity, or coupling, of all the metabolic reactions and corresponding genes within the cell.

Currently, numerous *E*. *coli* metabolic networks have been constructed for commensal, enterohemorrhagic, and extra intestinal pathogenic strains [1,4]. Unlike studies using *E*. *coli* metabolic models, a *Salmonella* Typhimurium LT2 metabolic model was used to examine metabolic reactions and the corresponding essential genes that are necessary for cell viability during the infection process under simulated conditions inside the host [9]. The evolutionary process that leads to genome changes is based on the theory of natural selection, which states that in a given environmental niche, there is constant pressure to retain genes that are important for growth and survival in that particular condition. When the availability of nutrients in a host-cell environment can be used to further define the mathematical constraints for the metabolic model mimicking host-cell nutrient environment, a technique termed flux balance analysis (FBA) was used that identified 417 reactions used by *S*. *typhimurium* LT2 during human infection [9].

To systematically explore genes predicted as essential and important for cell growth in a given environment, we used an approach that focused on three main components: 1) generating a metabolic network and corresponding metabolic model representing the metabolic capabilities of the *E*. *coli* pangenome which contains the union of all genes that encode metabolic reactions from 16 genomes of *E*. *coli*, 2) using flux balance analysis to systematically test growth predictions in three simulated host environments of all single gene mutants, and 3) comparing the essential/important gene predictions (i.e. those that promote growth and would likely have been retained over time) with sequenced enterobacterial genomes to determine if these genes were retained or lost in modern day strains.

In this work, we have developed new methods using constraint-based optimization and metabolic model construction to identify genes important for growth/survival in environments simulating three locations within the human body and have compared the predictions with actual evolutionary outcomes of sequenced genomes of enterobacterial pathogens, such as extraintestinal *E*. *coli*, that cause human disease in locations other than the intestinal tract. Extraintestinal *E*. *coli* infections may result in serious illness and even death, and globally 130–175 million cases of urinary tract infections are caused by Extraintestinal *E*. *coli* [10]. The urinary tract is also the most common route for *E*. *coli* causing bloodstream infections, which cause more than 40,000 deaths from septicemia each year worldwide [10]. Therefore, an understanding of the genes that are essential for the growth of these pathogens to survive in certain human body niches is of great interest to aid efforts on developing new control strategies and therapeutics.

Computational modeling allows us to conduct experiments of disease-causing bacteria where actual testing in humans is not an option. These are the three main objectives that were investigated: i) Can different locations in the human body be modeled using constraint-based linear programming? ii) Are there different predictions of essential/important genes for growth in simulated conditions representing three human body locations? iii) Do these gene predictions correlate with the genome content of modern-day enterobacterial pathogens that actually cause disease in each of the three locations? Overall, this study illustrated that mathematical constraints can be used with metabolic models to simulate the nutrient conditions the pathogen encounters during the infection process, and the genes predicted using FBA with the metabolic model simulating conditions during infection correlate with transcriptional gene-expression data obtained for conditions representing host-pathogen interactions. The central hypothesis is that the essential and important genes for bacterial growth in certain environments should be mostly remained over time in the genome of strains that cause disease in the corresponding human body locations, whereas the loss of those essential and important genes should not cause dire consequence for strains that invade different human locations.

## Results and Discussion

### Computational simulation of different niches in the human body

For the three simulated conditions, analytical data were used to add constraints that dictate metabolite availabilities respectively under three simulated conditions, the human macrophage cell [9], the bloodstream [11], and the urinary tract [12]. During the macrophage invasion, the pathogens can be engulfed and chained inside the pathogen-containing vacuoles that may restrict nutrients for cell growth. There is very little information on the nutrient compositions of those vacuoles under different macrophage activation states. Considering the pathogens may achieve nutrients from cytoplasm by modifying the membrane of vacuoles, existing literature values on the nutrient composition of the macrophage cytoplasm can be used to mimic the environment inside a macrophage for pathogen growth.

For the three simulated niches examined in human body, there were 15 available metabolites used as constraints shared in common for all three host niches, whereas 51 metabolites varied depending on the environment, indicating that differences in human body locations lead to different metabolite compositions available to the microrganisms (Table 1).

**Table 1:** Nutrients used to simulate three host environmental conditions.

Metabolites	Macrophage	Blood	Urine
2-Oxoglutarate	-	+	-
Acetoacetate	-	+	-
Adenine	-	-	+
Adenosine	-	+	-
Allantoin	+	+	+
Arabinose	+	-	-
Butyrate	-	+	+
Carnitine	+	-	-
Citrate	-	+	+
Cytosine	+	-	-
Deoxycytidine	+	-	-
Ethanolamine	+	-	+
Formate	-	-	+
Fructose	+	-	-
Fucose	+	-	-
Fumarate	-	+	-
Galactarate	+	-	-
Galactonate	+	-	-
Glucarate	+	-	-
Gluconate	+	-	-
Glucosamine	-	+	-
Glucose	+	+	+
Glucuronate	+	+	+
Guanine	-	-	+
Hypoxanthine	+	-	-
Inosine	+	-	-
D-lactate	-	+	+
L-lactate	-	+	+
L-Malate	-	+	-
D-Malate	-	+	-
Maltose	+	-	-
Mannitol	+	-	-
Mannose	+	-	-
Melibiose	+	-	-
Myo-Inositol	-	+	+
N-Acetyl-D-glucosamine	+	-	-
N-Acetylneuraminate	+	-	-
Nicotinate	-	+	-
Pantothenate	+	-	-
Propane-1,2-diol	+	-	-
Putrescine	+	-	-
Pyruvate	-	+	+
Rhamnose	+	-	-
Ribose	+	-	-
Sorbitol	+	-	-
Spermidine	+	-	-
Succinate	-	+	-
Taurine	-	-	+
Thiamin	+	+	-
Uracil	+	-	-
Uridine	+	-	-

Present / Not Present = + / -

### Predictions of essential/important genes for cell growth in three simulated human body locations

**Fig 1:**
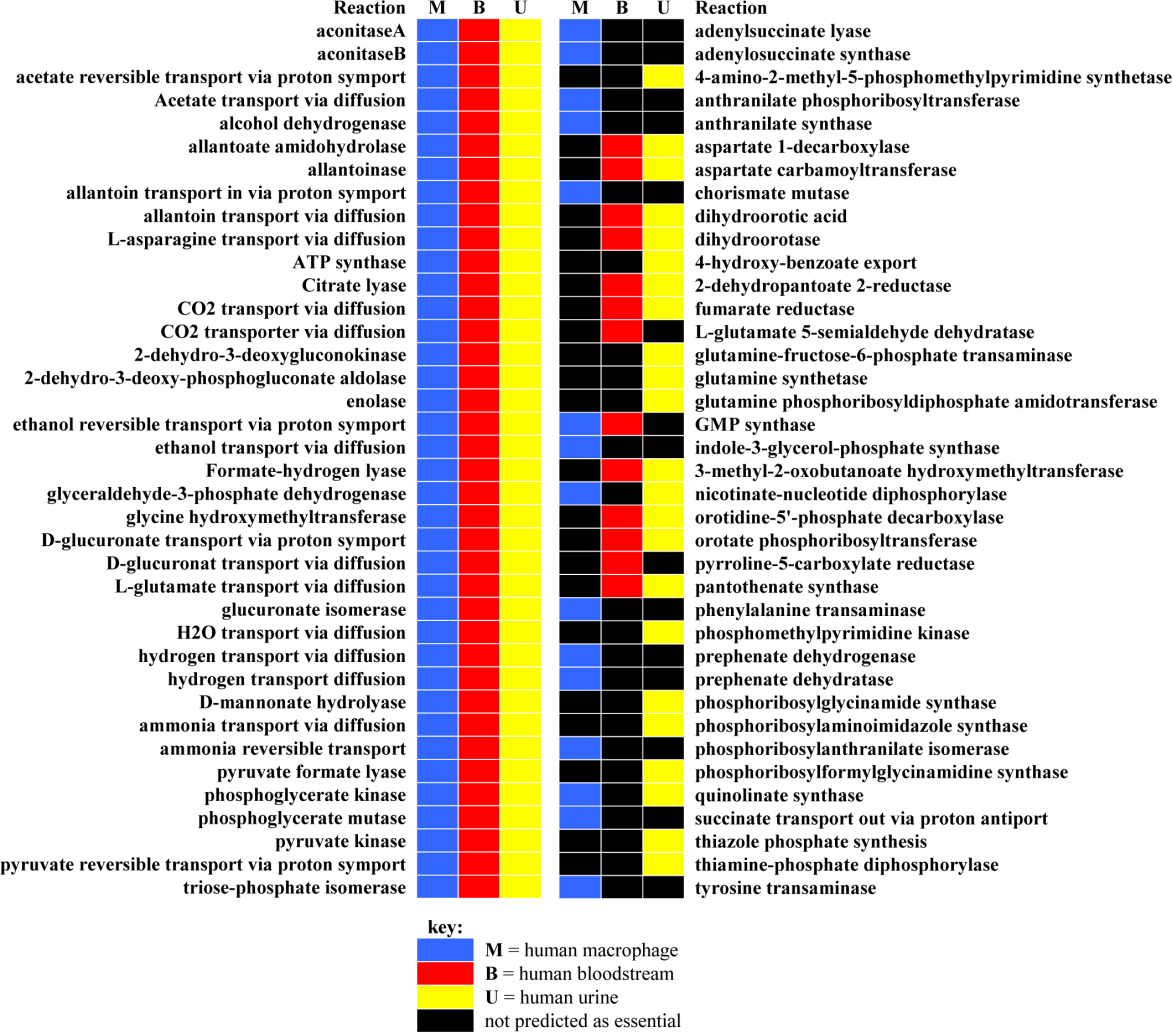
Essential reactions predicted for three simulated host environmental conditions. There are 38 reactions predicted to be commonly essential for all three simulated human body locations, whereas 38 essential reactions predicted that are differed for simulations of the human bloodstream, urinary tract, and macrophage.

When FBA analysis for single reaction deletions and their corresponding genes was conducted in the three simulated environments, the results varied in the total number of predicted essential and important reactions and associated genes for each condition (Table 2). Following each gene deletion, if the rate of biomass production was calculated as a value of zero (no growth prediction) or a reduction of >1% of the wild type biomass production, the genes were considered to be essential or important, respectively. There were 38 reactions predicted to be commonly essential for all three simulated human body locations, as the absence of them led to no cellular growth (Fig 1). Besides, 38 reactions were predicted as essential that were not shared in common for those conditions (Fig 1). There was only one reaction predicted to be important that resulted in a decrease of predicted biomass for all three simulated host locations, whereas 121 reactions were predicted as important that led to a predicted biomass reduction in one or two simulated conditions (S1 Data). For all of these essential and important reactions the genes correspond to, the reactions were identified to report the number of essential or important genes’ lost (S2 Data).

**Table 2:** Total number of reactions and corresponding genes predicted as essential and important for growth in three simulated human body locations.

Host niche	Essential reactions	Important reactions	Essential genes	Important genes
Macrophage	195	146	290	146
Bloodstream	193	65	288	182
Urinary tract	203	52	304	151

### Comparison of essential/important gene predictions based on the genomes of real disease-causing enterobacterial pathogens in each of the three host niches

Once the essential and important genes were identified, they were compared with the sequenced genomes of enterobacterial pathogens that invade the macrophage cell, infect the bloodstream, or cause disease in the urinary tract. Three genomes (*E*. *coli* UTI89, *E*. *coli* 53638, and *Salmonella* LT2) were used for essential and important gene comparison, and the genome of *E*. *coli* O157:H7 was used as a control because of the pathogen’s capability to cause disease in the human intestine. *E*. *coli* UTI89 is able to infect the urinary tract or the bloodstream in human body, causing disease outside the intestinal track. Both *E*. *coli* 53638 and *Salmonella* LT2 can cause disease by invasion of a host cell (Table 3).

**Table 3:** *E*. *coli* and *Salmonella* genomes used in this study.

Host niche	Enterobacterial human pathogenic strains	Genome of strain that causes disease
Bloodstream	Extraintestinal pathogenic *E*. *coli*	*E*. *coli* UTI89
Macrophage	*Salmonella* spp., Enteroinvasive *E*. *coli*	*E*. *coli* 53628, *Salmonella* LT2
Urinary tract	Urinary tract pathogenic *E*. *coli*	*E*. *coli* UTI89
Intestinal tract (control)	Enterohemorrhagic *E*. *coli*	*E*. *coli* EDL933

The central hypothesis is that the pathogens that actually cause disease in a given host location should have lost the fewest number of essential and important genes predicted for that conditions simulated *in silico* (macrophage, bloodstream, or urinary tract). In contrast, the pathogenic *E*. *coli* O157:H7 that causes disease in the intestinal tract would most likely have lost the most number of essential and important genes predicted for each of the three host niches. The host niche condition was not simulated for the control in this project. As shown in Table 4, when compared to the genomes of these organisms, the number of lost essential and important genes in each strain varied. When the numbers of both lost predicted essential and important genes out of the total number are summarized (Table 5), it is clear that some of the predictions match the real evolutionary outcomes of the genome content of these organisms, whereas the simulation of the urinary tract did not match the evolutionary outcomes of these strains, and this discrepancy is addressed in the conclusions section.

**Table 4:** Total number of predicted essential and important genes lost out of total predicted for each strain.

Host Niche	Genes Lost/Total Predicted	*E*. *coli* 53638	*E*. *coli* UTI89	*Salmonella* LT2	*E*. *coli* O157:H7
Macrophage	essential genes	2/290	4/290	3/290	13/290
Macrophage	important genes	20/366	17/366	22/366	58/366
Bloodstream	essential genes	2/288	1/288	3/288	12/288
Bloodstream	important genes	14/182	12/182	18/182	26/182
Urinary tract	essential genes	2/304	6/304	4/304	12/304
Urinary tract	important genes	9/151	11/151	15/151	19/151

**Table 5:** Total number of predicted essential and important genes lost out of total predicted for each strain.

Host niche	*E*. *coli* 53638	*E*. *coli* UTI89	*Salmonella* LT2	*E*. *coli* EDL933 (control)
Macrophage	22/656^c^	21/656^c^	25/656^c^	71/656^a^
Bloodstream	16/470^a^	13/470^a^	21/470^a^	38/470^a^
Urinary tract	11/455^b^	17/455^b^	19/455^b^	31/455^a^

^a^Evolutionary outcome agrees with *in silico* predictions for genome content

^b^Evolutionary outcome disagrees with *in silico* predictions for genome content

^c^Evolutionary outcome is within standard deviation with *in silico* predictions for genome content

## Conclusions

This study investigated *in silico* metabolic modeling and prediction of genes required for growth and survival in three human body locations. Based on the numerous differences of metabolites present in three different human body niches, this study illustrates that multiple environmental niches in a human can be simulated to study microbial metabolism by using constraint-based linear programming and computational model. Simulation of these three conditions led to different predictions of essential and important genes/reactions, which match the real evolutionary outcomes when compared to the control genome of the intestinal pathogen enterohemorrhagic *E*. *coli* O157:H7 strain EDL933, a strain that causes disease in the intestine and was predicted to have lost the most of the essential or important genes in the three other host niches. In the case of intracellular invasion, although the strain isolated from a urinary tract infection has the fewest essential/important genes lost, the two genomes of strains that actually cause disease through this route had very similar low numbers of lost essential/important genes. In the case of the simulations for the human bloodstream and urinary tract, *E*. *coli* UTI89 is the strain that actually causes disease in these locations, and had the least amount of necessary/important genes lost, which agreed with the evolutionary outcome. This project demonstrated that human body environments such as the bloodstream can successfully lead to accurate predictions of essential/important genes using optimization and constraint-based metabolic techniques. The discrepancies from the predictions for the urinary tract may indicate that more information is required for additional constraints to more accurately simulate this environment, or that the *E*. *coli* strains that have been characterized as causing disease in only one niche in the human body may also be capable of causing disease in numerous locations in the human body. Overall, this project was a success and lays a foundation towards future work to model metabolism of pathogenic microbes in different locations inside a human host. Since the actual infection study of these organisms in human is not a possibility, computer modeling of related disease processes becomes an emerging approach and field that is likely to grow immensely. By addressing these research ideas revealed by this project using optimization and constraint-based linear programming, the field of microbial system biology can be furthered to efficiently examine genome evolution.

## Materials and Methods

### Pangenome Metabolic Network Reconstruction

The metabolic model representing the *E*. *coli* pangenome (iEco1712_pan) used in this work was previously reconstructed based on the gene to protein to reaction (GPR) information of 16 *E*. *coli* genomes obtained from the ASAP database [1]. Draft and complete genomes have been continually updated using new publicly accessible genomes in the ASAP database since its inception [13]. There currently are 39 genomes among more than 150 enterobacteria genomes in the ASAP database that belong to *E*. *coli*, of which 16 are completely finished and were used in the construction of the metabolic model of *E*. *coli* pangenome (iEco1712_pan) [1]. The reconstructed network contains metabolic enzymes present in a union of 76,080 Open Reading Frames (ORFs) that map 17,647 Clusters of Orthologous Groups (COGs), with each ORF being assigned to an COG in the ASAP database, and all of the information for model composition, GPR associations for the *E*. *coli* pangenome (iEco1712_pan) reconstruction used in this work are available as supplemental information along with the sbml file for the iEco1712_pan GEM [1].

### Flux Balance Analysis

Flux balance analysis (FBA) has been commonly applied for mathematical analysis of GEMs, which can predict reactions-related fluxes in a metabolic network [14]. By constraining fluxes with steady-state mass balances, reaction directionality, and metabolite availability, a range of possible flux values can be generated in FBA. An objective function then can be used to identify flux distributions that maximize (or minimize) the objective function with those constraints. Biomass production, a commonly used objective function for FBA performance and for a proxy of growth, was adapted in this study [15]. FBA was conducted using the software package GAMS in this study, in which the *E*. *coli* pangenome metabolic network is described as a stoichiometric matrix (**S**_**i,j**_) with rows (iЄI) representing the metabolites and columns (jЄJ) indicating reactions that correspond to genes (gЄG). In a steady-state, the mass balance equation can be described as below, with *v* being the flux vector. Additional constraints are showed as lower and upper limits for the values of fluxes through reactions in a network.

$$\begin{array}{c} Max\;Vbiomass\\ \begin{array}{c} \text{s}.\text{t}.\;{\text{S}}_{\text{ij}}●\text{v}=0 \end{array}\\ v_{j,lb}<v_{j}<v_{j,up} \end{array}$$ (1)

The matrix built for *E*. *coli* pangenome GEM contains 1,726 metabolites (I) and 2,324 reactions (J) that associate with 1,712 genes (G). Three different niches located in the human body (macrophage, blood, and urinary tract) were simulated to set constraints for FBA in this study, with possible metabolite compositions being identified through literature review that determined analytical compositions of nutrients present in each bodily location. The simulated condition for macrophage contains 32 metabolites, the bloodstream environment contains 19 metabolites, while there are 14 metabolites that belong to the urinary tract niche (Table 1).

### Gene Essentiality

**Fig 2:**
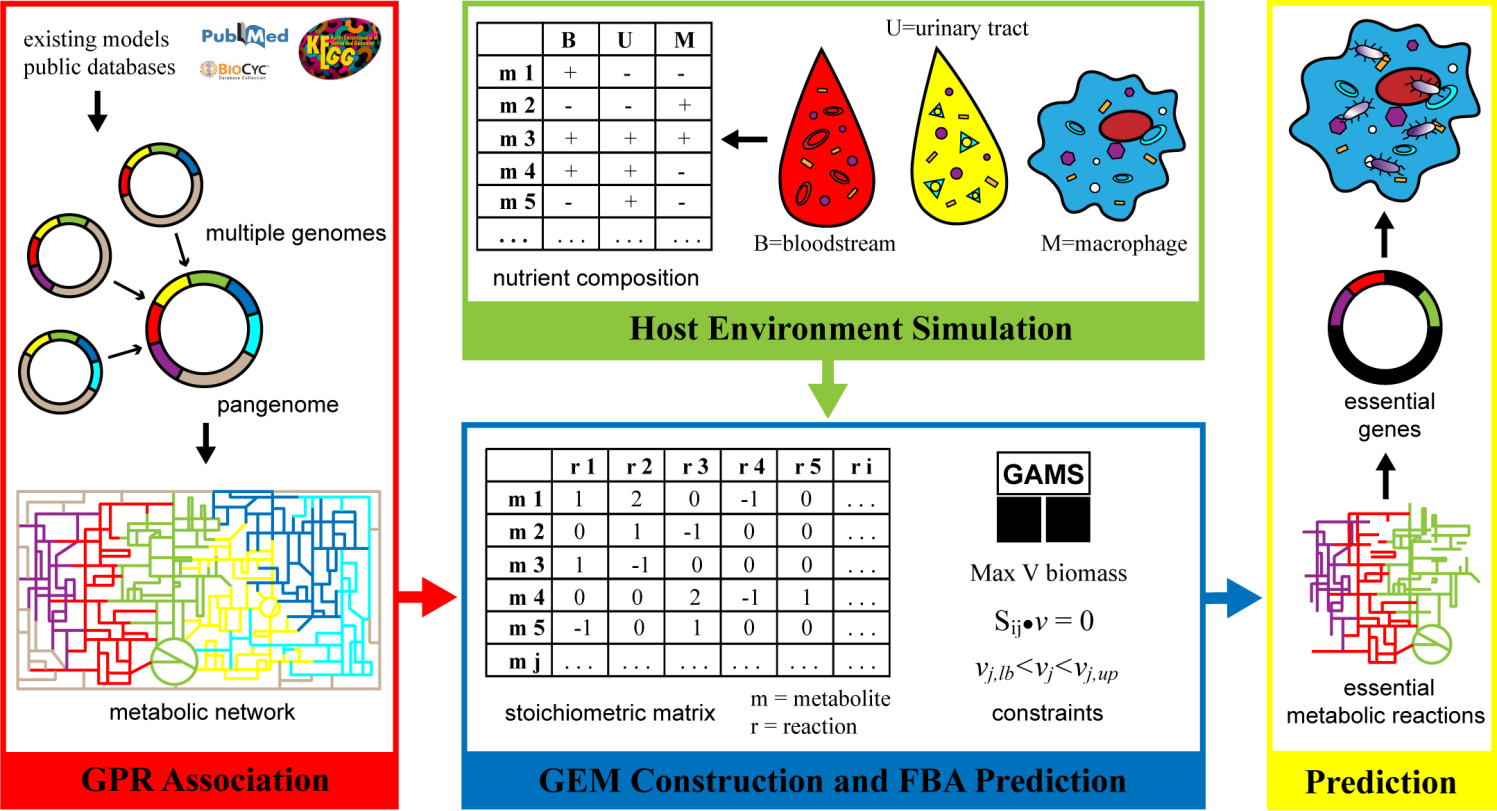
Essential gene identification using GEMs predictions under simulated environment. The GEM constructed upon pangenome incorporated from *E*. *coli* genomes can be used to generate predations with simulated nutrient conditions to identify essential genes along with corresponding essential metabolic reactions under multiple human body niches.

Unlike virulence factor genes [16], essential genes are those required to maintain critical cellular functions under specific environments, while important genes are not irreplaceable but still necessary for robust bacterial growth under those conditions. To determine the essentiality of genes expressed under different environmental pressures (macrophage cell, bloodstream, and urine tract), genes were removed one-by-one in networks and the resulting changes in biomass production rate can be estimated to reveal the impact of gene loss (a proxy for fitness). Following each gene deletion, if the calculated value of biomass production rate was zero, meaning no predicted intracellular growth, the gene would be considered essential. Important genes were predicated based on >1% reduction of the wild type biomass production rate. A graphic description on identifying essential genes and corresponding metabolic reactions using GEMs constructing and computational predictions is showed in Fig 2.

## Supporting Information

S1 Data Reactions predicted as important for all three simulated environmental conditions and that differed during simulation of the human bloodstream, urinary tract, and macrophage. (XLSX)

S2 Data Reactions corresponding to essential gene predictions for the *E*. *coli* pangenome GEM. This file contains three tables, the first contains all predicted essential reactions during simulation of human macrophage, the second contains all human bloodstream predicted essential reactions, and the third contains predicted essential reactions during simulation of the human urinary tract.
(XLSX)
